# Consumption of a Specially-Formulated Mixture of Essential Amino Acids Promotes Gain in Whole-Body Protein to a Greater Extent than a Complete Meal Replacement in Older Women with Heart Failure

**DOI:** 10.3390/nu11061360

**Published:** 2019-06-17

**Authors:** Il-Young Kim, Sanghee Park, Ellen T. H. C. Smeets, Scott Schutzler, Gohar Azhar, Jeanne Y. Wei, Arny A. Ferrando, Robert R. Wolfe

**Affiliations:** 1 Department of Geriatrics, the Center for Translational Research in Aging & Longevity, Donald W. Reynolds Institute on Aging, University of Arkansas for Medical Sciences, Little Rock, AR 72205, USA; SPark@uams.edu (S.P.); SESchutzler@uams.edu (S.S.); AzharGohar@uams.edu (G.A.); WeiJeanne@uams.edu (J.Y.W.); AFerrando@uams.edu (A.A.F.); RWolfe2@uams.edu (R.R.W.); 2 Department of Molecular Medicine, Lee Gil Ya Cancer and Diabetes Institute, Gachon University School of Medicine, Incheon 21999, Korea; 3 Department of Human Nutrition, Wageningen University, P.O. Box 17, 6700 AA Wageningen, The Netherlands; ellen.smeets@hotmail.com

**Keywords:** anabolic response, protein quality, sarcopenia, aging, stable isotope tracer

## Abstract

Heart failure in older individuals is normally associated with a high body mass index and relatively low lean body mass due to, in part, a resistance to the normal anabolic effect of dietary protein. In this study we have investigated the hypothesis that consumption of a specially-formulated composition of essential amino acids (HiEAAs) can overcome anabolic resistance in individuals with heart failure and stimulate the net gain of body protein to a greater extent than a commercially popular protein-based meal replacement beverage with greater caloric but lower essential amino acid (EAA) content (LoEAA). A randomized cross-over design was used. Protein kinetics were determined using primed continuous infusions of L-(^2^H_5_)phenylalanine and L-(^2^H_2_)tyrosine in the basal state and for four hours following consumption of either beverage. Both beverages induced positive net protein balance (i.e., anabolic response). However, the anabolic response was more than two times greater with the HiEAA than the LoEAA (*p* < 0.001), largely through a greater suppression of protein breakdown (*p* < 0.001). Net protein accretion (g) was also greater in the HiEAA when data were normalized for either amino acid or caloric content (*p* < 0.001). We conclude that a properly formulated EAA mixture can elicit a greater anabolic response in individuals with heart failure than a protein-based meal replacement. Since heart failure is often associated with obesity, the minimal caloric value of the HiEAA formulation is advantageous.

## 1. Introduction

Heart failure is a global public health concern, affecting approximately 6%–10% of individuals over the age of 65 worldwide [1]. The risk of death is 35% in the first year after diagnosis, and about 10% per year thereafter [2]. There are different types of heart failure, and a wide range of potential causes, including the natural process of aging [3]. Regardless of the specific underlying cause or type of heart failure, there are common pathophysiological responses. Most prominently, impaired exercise capacity, shortness of breath, fatigue and decreased muscle strength are hallmarks of heart failure that lead to decreased physical function, and ultimately to sedentary behavior and development of insulin resistance [4]. There are multiple reasons for impaired physical functional capacity in heart failure. Most attention has focused on the inability of the heart to pump an adequate amount of blood, with a consequent limitation in the delivery of substrates and oxygen to the skeletal muscles. However, drugs that target cardiovascular function have often failed to influence exercise capacity [5]. It is becoming clear that adverse responses in muscle directly impair physical function in individuals with heart failure. Physical training can improve exercise tolerance without affecting central hemodynamic function [6]. Testosterone treatment, which enhances skeletal muscle function while not affecting heart function, has also been shown to improve physical function in heart failure [7]. Thus, whereas impaired pumping action of the heart is central to limited physical capacity in heart failure, factors associated with skeletal muscle also play a role. 

Heart failure induces a loss of muscle mass and strength [4,8]. The loss of muscle mass is often not evident since many heart failure patients are overweight or obese, and obesity may be accompanied by an increased lean body mass [9]. Nonetheless, loss of muscle mass and strength in overweight elderly is common and has become recognized as “sarcopenic obesity” [10], wherein a deficiency in muscle mass and strength is particularly debilitating owing to the large body mass that must be moved. 

The underlying mechanisms responsible for loss of muscle in heart failure are uncertain. The results of studies of protein turnover in muscle and at the whole-body level have been inconsistent. There may be a greater loss of body protein in the post-absorptive state in some individuals with heart failure as compared to healthy elderly [11], driven largely by an increased rate of protein breakdown [12]. However, the magnitude of the basal response of protein kinetics in heart failure is insufficient to fully explain the extent of muscle loss. A diminished response to the normal anabolic effect of nutrient consumption (anabolic resistance), particularly with regard to dietary protein, may be an important aspect of the loss of muscle mass in heart failure. Limited responsiveness to the anabolic response to dietary protein is common to all forms of cachexia [13], as well as being a natural process of aging [14]. 

A nutritional solution to the problem of loss of muscle mass in heart failure, even in obese individuals, would be of great value. Ideally, such a composition would not only stimulate the net gain of body protein, but would do so with minimal caloric intake. Heart failure is commonly associated with obesity, and a nutritional formulation that adds significantly to caloric intake would be undesirable. Consumption of formulations of essential amino acids (EAAs) have been proven to overcome anabolic resistance in healthy older individuals (e.g., [15,16]). As few as 3 g of EAAs can elicit an effective anabolic response in healthy elderly [17], while reducing the potential burden of the production of by-products of the metabolisms of non-essential amino acids (NEAAs), such as urea and ammonia. The decrease in NEAAs occurs because of greater reincorporation into body proteins [18]. Further, the caloric value of small amounts of EAAs is inconsequential, as compared to a significant number of calories in most meal replacement beverages. Nonetheless, the use of traditional meal replacement beverages to promote anabolism in older adults far exceeds the use of EAA formulations.

In the current study we have assessed the anabolic response in individuals with heart failure by means of the measurement of whole-body protein synthesis and breakdown. We have tested the hypothesis that a low-calorie EAA-based dietary supplement will induce a significantly greater gain in whole-body net protein balance than a conventional meal replacement beverage specifically targeting support of heart failure patients. The comparison is significant not only in terms of the responses of protein synthesis and breakdown to a conventional serving, but also because of the considerable differences in the caloric intake associated with the two nutritional approaches. 

## 2. Materials and Methods

### 2.1. Subjects

Eight elderly (80 ± 7 years) women with heart failure participated in this randomized cross-over study. Heart failure was defined as New York Heart Association (NYHA) class I or II. Subjects took at least one of the following classes of medications: [1] angiotensin converting enzyme inhibitor (ACE inhibitor), [2] β-blocker, [3] diuretics, or [4] angiotensin II receptor blockers. Exclusion criteria were anemia (hemoglobin: <10.0 g/dL), history of a chronic inflammatory condition or disease, myocardial infarction in the past 6 months, unstable angina, moderate to severe valvular heart disease, significant ventricular arrhythmias, moderately severe dementia (mini-mental state examination score: <18), history of malignancy in the past 6 months, cancer chemotherapy or radiation within 1 year, hemoglobin A1c >9.0%, insulin usage for diabetes control, chronic kidney disease (glomerular filtration rate: <30 mL/min), more than 5% body weight loss during the past 6 months, or documented infiltrative, restrictive or hypertrophic cardiomyopathy, and allergy to milk or milk-based products. One subject was excluded from the final analysis of whole-body protein kinetics because of problems with the measurement of isotope enrichment. All subjects gave their written informed consent and the study was approved by the Institutional Review Board at the University of Arkansas for Medical Sciences.

### 2.2. Experimental Design

The experimental protocol determined the response of whole-body protein kinetics to a single dose of one of two beverages in the basal state and for four hours after consumption. A cross-over experimental design was used in which each subject consumed both beverages on separate occasions. During the screening for subject eligibility, body composition was determined by dual-energy X-ray absorptiometry (DEXA, QDR-4500A; Hologic, Waltham, MA, USA). Once accepted into the study, subjects completed two 7-h metabolic tracer infusion trials: the first 3-h time period (0–180 min) for determination of basal, post-absorptive protein kinetics, and the last 4-h time period (180–420 min) for determination of whole-body anabolic response to a specially-formulated composition of EAAs drink (HiEAA; The Amino Company, LLC, Los Angeles, CA, USA) or a commercially popular protein-based meal replacement beverage with greater caloric but lower EAA content (LoEAA; Ensure Active Heart Health, Abbott Nutrition, Columbus, OH, USA). Subjects consumed the beverages within <5 min starting at 180 min. The order of beverage consumption was randomized.

### 2.3. Test Drinks

Ensure Active Heart Health drink: A single serving of Ensure Active Heart Health chocolate drink (Abbott Nutrition) of 237 mL (one bottle) consisted of 8 g of protein in the form of milk protein isolate and soy protein isolate. The amount of each protein was proprietary and not specified, so the exact amino acid composition was uncertain, but was approximately 4 g of EAAs per serving. Additionally, the drink contained approximately 3 g of fat and 21 g of carbohydrates. The caloric value of one serving was 140 kcal. 

AA drink: The HiEAA formulation consisted of the following amino acids: histidine (1.6%), isoleucine (9.6%), leucine (34%), lysine (13.6%), methionine (2.7%), phenylalanine (5.5%), threonine (6.8%), valine (6.8%), tryptophan (2.7%), and citrulline (10.9%), as well as flavoring (total calories = 48 kcal). The HiEAA drink was made by dissolving 15 g of the powdered formulation (containing 12 g amino acids plus 3 g of flavoring) in 240 mL of distilled water.

### 2.4. Stable Isotope Tracer Infusion Protocol 

We quantified the anabolic response to consumption of each beverage by determining whole-body protein kinetics (protein synthesis, protein breakdown, and net protein balance, g protein∙240 min). This approach not only quantifies changes in net protein balance at the whole-body level, but also reflects changes in net balance in response to nutrient intake by the muscle [19], without the invasiveness of femoral arterial and venous catheterization required to directly measure muscle net protein balance [20]. We chose to not measure fractional synthetic rate of muscle protein because it does not provide information on protein breakdown (and thus net balance), and accelerated protein breakdown has been reported to be the basis for muscle loss in heart failure [11,12]. The whole-body protein kinetic approach enables dynamic quantitation of all factors relevant to determining the magnitude of an anabolic response to nutrient consumption. 

**Figure 1:**
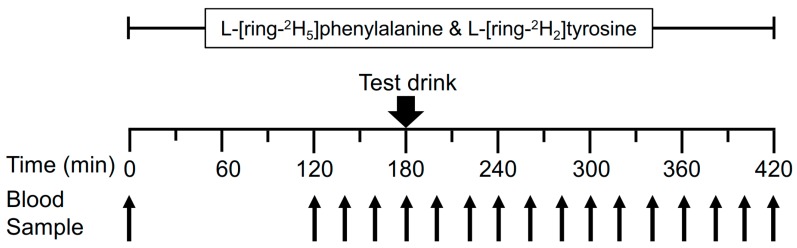
Experimental protocol. Each subject was studied twice. In one study the test drink was a specially-formulated composition of essential amino acids (HiEAA), and in the other study the test drink was a commercially available drink with lower essential amino acid content (LoEAA).

We used the phenylalanine (Phe)/tyrosine (Tyr) model to determine whole body protein kinetics [21]. The 7-h stable isotope infusion protocol is depicted in Figure 1. Subjects reported to the Reynolds Institute on Aging (RIOA) after an overnight fast (>8 h fasted after 2200). Before initiation of tracer infusion, subjects had catheters inserted into one lower arm for the tracer infusion and into a hand vein in the other arm for “arterialized” blood sampling using a heating box. A baseline blood sample was collected before the start of the tracer infusion to determine background isotopic enrichments and blood chemistry. Then, primed continuous infusions of L-(ring-^2^H_5_)phenylalanine (prime, 3.92 μmol/kg; infusion rate, 4.60 μmol/kg/h) and L-(ring-^2^H_2_)tyrosine (prime, 1.57 μmol/kg; infusion rate, 0.95 μmol/kg/h) and a priming dose of L-(ring-^2^H_4_)tyrosine (0.33 μmol/kg) were conducted for determination of whole-body protein kinetics (tracers were purchased from Cambridge Isotope Laboratories, Andover, MA, USA). Blood samples were taken in the fasted state (0, 120, 140, 160, and 180 min) and throughout the drink-fed state (200, 220, 240, 260, 280, 300, 320, 340, 360, 390, and 420 min) to measure tracer enrichment and plasma amino acid concentrations.

### 2.5. Analytic Methods

**Figure 2:**
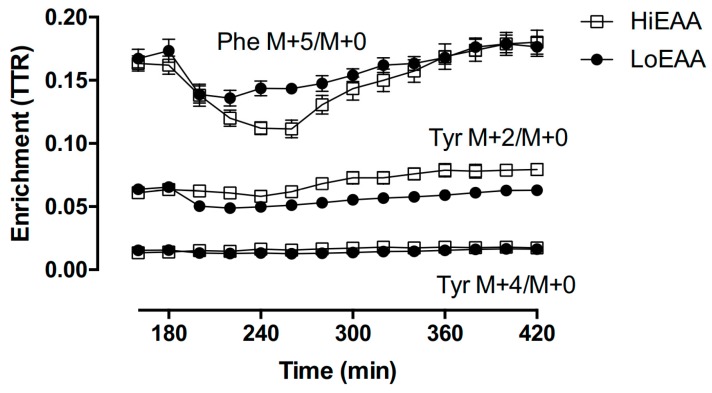
Plasma enrichments of infused tracers (Phe M+5/M+0 and Tyr M+2/M+0) and one (Tyr M+4/M+0) derived from Phe M+5 through hydroxylation. Samples were taken before and following consumption either the HiEAA or the LoEAA after the 180 min sample was collected. Values (*n* = 7) are expressed as mean ± standard error of the mean (SEM). TTR, tracer to tracee ratio; M+0 is the most abundant form of naturally occurring Phe or Tyr; M+*i* (e.g., M+2, M+4, and M+5) is heavier than M+0 by *i* mass unit.

Plasma samples were processed for determination of isotopic enrichment (Figure 2) and amino acid concentrations as previously described [22,23,24]. Tracer enrichments and plasma amino acid concentrations were determined by using liquid-chromatography mass spectrometry (QTrap 5500 MS; AB Sciex, Foster City, CA, USA) as previously described [23].

### 2.6. Calculations of Protein Kinetics

Protein kinetics (protein synthesis, breakdown, and net balance) following the HiEAA or the LoEAA were calculated as described previously [21,22,23,24] and expressed as changes from the basal, post-absorptive state to the fed state as previously described. Briefly, the calculation of protein breakdown in the post-absorptive state is based on the rate of appearance of Phe determined by traditional tracer dilution methodology, since Phe is not produced in the body. Protein synthesis is calculated as the difference between protein breakdown and the irreversible loss of Phe, determined as the rate of hydroxylation of Phe to Tyr. Calculation of protein kinetics in the post-prandial state requires accounting for the contribution of the dietary Phe to the total rate of appearance of Phe in the blood [25]. This involves an assumption regarding the true ileal digestibility [25]. Digestibility was assumed to be 100% for HiEAA and 95.3%, for the LoEAA, assuming protein digestibility of the LoEAA was similar to that of whey protein concentrate [26]. Account was taken of the amount of digested Phe that was cleared and metabolized in the splanchnic bed before reaching the peripheral circulation, from which blood samples were obtained. Irreversible loss of Phe in the splanchnic bed was determined from the measured rate of irreversible hydroxylation of Phe to Tyr, since the hydroxylation of Phe occurs entirely in the liver [25]. The splanchnic uptake of absorbed Phe was calculated by subtracting the amount of Phe hydroxylated in the post-absorptive state from the corresponding amount in the post-prandial state. In the current study the fraction of Phe uptake hydroxylated to Tyr was similar in the two groups, so the calculated value of splanchnic uptake of absorbed Phe did not affect the comparison of the responses between the two beverages. The total response of protein synthesis, protein breakdown, and net protein balance over the four hours after consumption of each of the beverages was calculated to minimize any uncertainties stemming from non-steady state calculations [27].

### 2.7. Statistical Analysis

A two tailed paired Student’s *t*-test was performed to compare differences between the drinks with respect to changes in whole-body protein synthesis, breakdown and net balance as compared to the post-absorptive basal state, total protein accretion, protein accretion efficiency (net gain in protein normalized for the amount of EAAs or calories consumed), and area under the curve of plasma amino acids. Two-way repeated measures of ANOVA were performed to compare differences in time-course responses of plasma amino acids followed by a two tailed paired *t*-test (if necessary). Statistical significance was declared with the *p*-values of <0.05. This analysis was using IBM SPSS Statistic Package software version 24 for Window (SPSS, Chicago, IL, USA).

## 3. Results

### 3.1. Baseline Characteristics

Baseline characteristics for all eight women are shown in Table 1. The mean age was 80 ± 7 years. Average body mass index was 32.7 ± 5.9 kg/m^2^, and body composition analysis by DEXA showed the subjects to have 45.5% ± 3.2% fat. The average systolic blood pressure was 145 ± 17 mm Hg, the mean diastolic blood pressure was 75 ± 5 mm Hg and the average heart rate was 73 ± 12 beats/min.

### 3.2. Whole-Body Protein Kinetics

**Figure 3:**
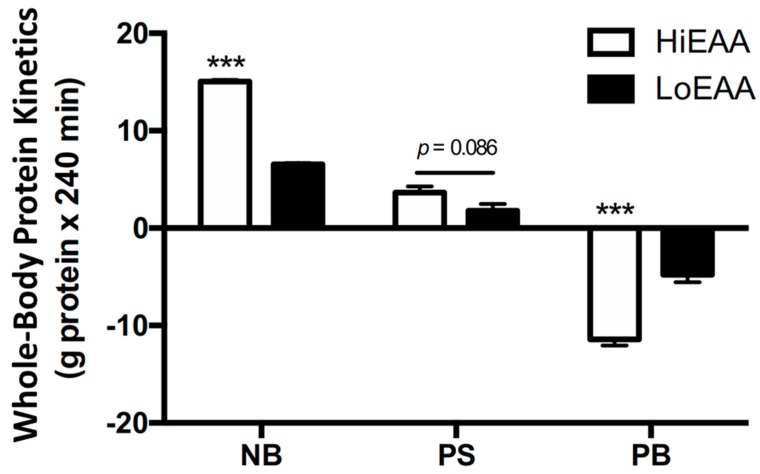
Total changes in whole-body protein kinetics (protein synthesis (PS), protein breakdown (PB), and net protein balance (NB)) from basal post-absorptive values following either the HiEAA or the LoEAA. *** Significantly different between meals, *p* < 0.001. Values (*n* = 7) are expressed as mean ± SEM.

In the fasted states, whole-body protein kinetics (expressed as mg protein/kg lean body mass (LBM)/min) were not different between the HiEAA and the LoEAA: net protein balance, −0.29 ± 0.04 and −0.31 ± 0.03; protein synthesis, 3.01 ± 0.11 and 2.90 ± 0.14; protein breakdown, 3.30 ± 0.10 and 3.21 ± 0.14 for the HiEAA and the LoEAA, respectively). Consumption of both HiEAA and LoEAA resulted in a positive net protein balance (HiEAA: −0.29 ± 0.04 to 1.50 ± 0.11; LoEAA: −0.31 ± 0.03 to 0.32 ± 0.05; (both, *p* < 0.001)) through a stimulation of protein synthesis (HiEAA: 3.01 ± 0.11 to 3.38 ± 0.17; LoEAA: 2.90 ± 0.14 to 3.09 ± 0.09 (both, *p* < 0.05)) and a suppression of protein breakdown (HiEAA: 3.30 ± 0.10 to 1.89 ± 0.12; LoEAA: 3.21 ± 0.14 to 2.7 ± 0.08, (for both, *p* < 0.001)), compared to basal, post-absorptive values. The magnitude of increase in net protein balance from the basal post-absorptive state (i.e., total anabolic response, expressed as g protein∙240 min) following feeding was greater with the HiEAA as compared to the LoEAA (*p* < 0.001) (Figure 3). The greater net protein balance with the HiEAA was largely due to suppression of protein breakdown (*p* < 0.001) with a marginally greater stimulation of protein synthesis as compared to the LoEAA, although a statistical significance in protein synthetic rates was not reached.

### 3.3. Protein Accretion Efficiency

**Figure 4:**
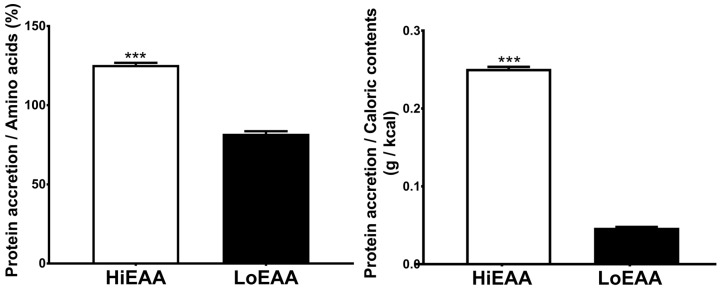
Net protein accretion efficiency. Efficiency was determined by dividing net protein accretion (g protein over 240 min) by either the amino acid content, multiplied by 100 (left) or by the caloric content (right) of the HiEAA or the LoEAA. *** Significantly different from the LoEAA, *p* < 0.001. Values (*n* = 7) are expressed as mean ± SEM.

The amount of amino acids contained in the respective drinks, as well as the caloric value of the beverages, were different between HiEAA (12 g amino acids (AAs)) and LoEAA (4 g AAs). To access efficiency of protein accretion, we normalized net protein accretion to the amount of amino acid intake (%), or to the amount of caloric intake (g/kcal). In both cases, protein accretion efficiency was significantly greater with the HiEAA compared to the LoEAA (for all, *p* < 0.001, Figure 4).

### 3.4. Plasma Amino Acid Responses

**Figure 5:**
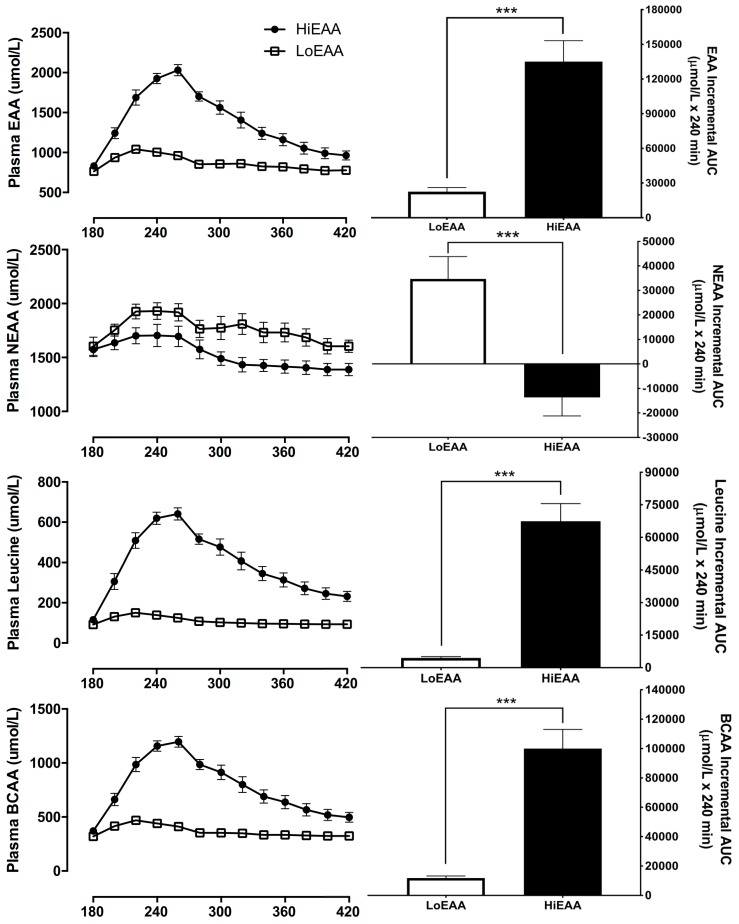
Time course responses and total area under curves (AUC) of plasma concentrations of essential amino acids (EAAs), non-essential amino acids (NEAAs), leucine, and branched chain amino acids (leucine, isoleucine, valine, BCAAs) following either consumption of the HiEAA or the LoEAA. There were significant effects for treatment, time, and treatment-by-time interaction for EAAs, leucine, and BCAAs (for all, *p* < 0.001) as well as NEAAs (for all, *p* < 0.02). Values (*n* = 8) are expressed as mean ± SEM. *** significantly different from the LoEAA, *p* < 0.001.

Responses of plasma concentrations of EAAs, NEAAs, leucine, and branched chain amino acids (BCAAs) following the HiEAA or the LoEAA are presented in Figure 5. There were significant effects for treatment, time, and treatment-by-time interaction for plasma responses of EAAs, leucine, and BCAAs (for all, *p* < 0.001) as well as NEAAs (for all, *p* < 0.02): EAAs, leucine, and BCAAs were significantly higher but NEAAs were lower with the HiEAA compared to the LoEAA (Figure 5).

## 4. Discussion

We have compared whole-body anabolic responses to consumption of a uniquely-formulated EAA beverage (HiEAA) with consumption of a commercially popular meal replacement beverage (LoEAA) in older female subjects with heart failure. Anabolic resistance is common in older adults, and the body composition of our subjects (45.5% fat) suggests that was likely the case in our subjects. Nonetheless, both beverages stimulated an anabolic response. However, consumption of the EAA-based beverage (i.e., HiEAA) resulted in a more than two-fold greater anabolic response (i.e., gain in net protein balance) than the commercially popular beverage (i.e., LoEAA) through a greater suppression of protein breakdown with a marginally greater stimulation of protein synthesis. Importantly, the net anabolic response normalized to either amino acid or caloric content of the beverages was also greater with HiEAA than LoEAA when net balance was expressed as an absolute value. 

The net anabolic response is determined by the balance between protein synthesis and protein breakdown, i.e., through stimulation of protein synthesis, suppression of protein breakdown, or a combination of the two [28]. Consumption of dietary protein or amino acids, either alone [15,29] or in the context of mixed meals, induces an anabolic response [21,22,23,24]. However, the aspects of protein kinetics that lead to an anabolic response following intake of dietary protein or amino acids may vary depending on the context of intake. For example, stimulation of protein synthesis drives the positive anabolic response following intake of “high-quality” protein or amino acids alone or as a part of protein food sources [15,29]. In contrast, in the context of more commonly consumed mixed meals, suppression of protein breakdown is quantitatively the main driver of the anabolic response [22,23,24,25]. In this regard it is striking that in the present study the HiEAA, which consisted of mainly EAAs, resulted in a greater anabolic response compared to the LoEAA, largely through a suppression of protein breakdown. Suppression of protein breakdown is important because accelerated protein breakdown is the primary basis for loss of body protein in heart failure [11,12].

The mechanism underlying the suppression of protein breakdown in response to HiEAA was not likely an insulin response, as we have previously shown that insulin concentration is not increased in response to EAA ingestion [14]. Further, the LoEAA formulation contained 21 g of carbohydrate, so a greater insulin response would be expected following consumption of the LoEAA as compared to the HiEAA. Previously, we proposed that a rise in intracellular EAA concentrations play an important role in inhibiting protein breakdown [28,30]. Thus, it is likely that the amount of EAAs contained in the HiEAA (~12 g) provided a strong signal for suppression of protein breakdown. Consistent with the notion, it was shown that intracellular amino acid concentrations only increased in response to an increasing rate of amino acid infusion when the maximal rate of muscle protein synthesis was achieved [31]. Furthermore, in previous studies in which the anabolic effect of amino acids was attributed only to protein synthesis [14,28], the dosage of EAA intake in most cases was less than in the current study. Also, several studies assessing the anabolic response to amino acids have not measured protein breakdown (e.g., [32]). Therefore, the most likely explanation of the suppression of protein breakdown in the HiEAA is that the dosage of EAAs or specific EAA component(s) was large enough to cause a significant increase in the intracellular amino acid concentrations.

It was impossible to balance all aspects of the servings of the two beverages. Equalizing the total amino acid content would have required a 50% increase in the amount of the LoEAA consumed, which would have significantly amplified the existing difference in caloric content, and there still would be a difference in EAA content between the two beverages. Conversely, reducing the amount of the LoEAA consumed to equal the caloric intake of the HiEAA beverage would have greatly amplified the difference in amino acid content. Consequently, we compared the responses to standard servings of the two beverages, as this was the most clinically-relevant comparison. In order to compare the beverages in terms of metabolic efficiency, we have expressed the results not only in terms of the total response to a standard dose, but we also normalized the results by either total amino acid content or total caloric content. Regardless of how the data were expressed, the response was greater to the HiEAA than the LoEAA. The greater efficiency of the HiEAA is striking, since the non-protein components of the LoEAA beverage would be anticipated to augment the anabolic response to the protein component. Co-ingestion of either carbohydrate [33] or fat [34] are well documented to amplify the anabolic response to amino acid or protein intake. The advantage of the HiEAA per calorie ingested is particularly clinically relevant. A nutritional approach to increasing lean body mass in heart failure that adds significantly to caloric intake is likely to be counter-productive. Heart failure is usually accompanied by obesity, as reflected by the BMI (32.7 ± 5.9) and body composition (45.5% ± 3.2 % body fat) of our subjects, so any additional caloric intake is undesirable.

There are several aspects of the HiEAA beverage that would be expected to confer an anabolic advantage as compared to the intact protein in the meal replacement, even considering the greater caloric value of the LoEAA. First, the maximal anabolic response requires all of the EAAs. Consumption of branched chain amino acids alone in heart failure had no benefit on muscle protein balance [13]. NEAAs are not required, as endogenous NEAAs were used to produce new proteins when the HiEAA were consumed. In contrast, the LoEAA beverage is more than half NEAAs by volume. We have previously shown that the addition of NEAAs to a mixture of EAAs provides no benefit in terms of protein synthesis [35].

In addition to the inclusion of all the EAAs and exclusion of NEAAs in the HiEAA beverage, free amino acid formulations have the advantage that the composition can match the metabolic requirements of specific circumstances. The HiEAA formulation contained approximately 34% leucine. We have previously reported that anabolic resistance in older adults can be overcome with consumption of a leucine-enriched EAA mixture [15]. The rationale for the high leucine content is that the normal activation of the eukaryotic initiation factors, most notably through mechanistic target of rapamycin (mTOR), is suppressed in the anabolic resistant state and that suppression can be overcome by the action of leucine to activate mTOR [36]. Additionally, the profile of the other eight EAAs in the formulation should increase the intracellular availability of each EAA in proportion to its relative abundance in muscle protein. To accomplish the optimal intracellular EAA concentrations, account was taken not only of the composition of muscle protein, but also the empirically-determined transport rates of the individual EAAs from blood into muscle [37]. Minimal amounts of histidine, tryptophan, and methionine were required, making greater proportions of the other EAAs possible. Finally, arginine becomes an essential amino acid in the older adults [38]. Arginine supports protein synthesis [39] as well as the production of nitric oxide [40]. Nitric oxide is responsible for regulation of blood pressure and regional blood flow [38]. While arginine supplementation can increase blood arginine levels to some extent, the effect is blunted by the high hepatic clearance of absorbed arginine before it ever reaches the peripheral circulation. Citrulline is the precursor of arginine in the body. When citrulline is absorbed from the gastrointestinal tract it is converted to arginine in the kidney, thereby increasing the peripheral arginine concentration to a greater extent than ingestion of the same amount of arginine [38]. The increase in arginine concentration after consumption of citrulline causes a rapid increase in nitric oxide production in older women with heart failure [40]. An increase in nitric oxide production coinciding with an increase in plasma EAA concentrations should help to distribute the ingested EAAs to muscle by increasing muscle blood flow.

The greater anabolic efficiency of the HiEAA has potential clinical implications. First, a greater anabolic response sustained over time will translate to an amelioration of the loss of muscle mass and function that commonly occurs in both aging and heart failure. Second, an EAA-based formulation places minimal stress on the kidney, because the production of two major by-products of amino acid metabolism (i.e., ammonia and urea) is limited because NEAAs, including glutamine and alanine, are reincorporated into protein at an accelerated rate rather than being oxidized [18]. Increased incorporation of endogenous NEAAs into protein was reflected in the current study by the lower NEAA concentrations following consumption of the HiEAA despite a greater N content than the LoEAA (Figure 5). The resulting impact on the production of ammonia and urea minimizes the metabolic burden on the kidneys, and this may be particularly relevant to individuals with heart failure who often have reduced renal function as compared to healthy older adults [41]. Finally, the HiEAA was able to stimulate a greater anabolic response with negligible caloric intake, as opposed to the conventional meal replacement (i.e., LoEAA). Excess body weight is a frequent complication of heart failure in older individuals—the average BMI of our subjects was greater than 32, and the average percent body fat was greater than 45%. Increased body weight is a principle determinant of immobility in older adults [42], and decreased mobility contributes to further loss of muscle mass and function. Further, in contrast to a conventional meal replacement, consumption of an EAA-based dietary supplement does not impair the anabolic response to the next meal [43].

There is ample evidence supporting the predictive value of acute anabolic responses. The acute anabolic response to consumption of a different EAA formulation was shown to increase strength and functional capacity in healthy older individuals when consumed twice per day for 16 weeks [16,44]. In addition, the acute stimulation of muscle protein synthesis by consumption of a single dose of EAAs in both young and old individuals translated to significant amelioration of the loss in functional capacity resulting from strict bed rest when consumed three times per day for either 10 (older individuals) or 30 (young individuals) days [19,45]. Thus, although the current study only demonstrated an acute metabolic advantage of the HiEAA formulation, it is likely that over time regular consumption would result in beneficial functional responses.

## 5. Conclusion

We conclude that a recommended dose of the HiEAA effectively stimulated an anabolic response in older females with heart failure. A single serving of a popular meal replacement targeting individuals with heart failure (Ensure Active Heart Health, LoEAA) also induced an anabolic response, but the response to the HiEAA was approximately 2.5-fold greater. The anabolic response of the HiEAA was also greater when normalized for the amino acid and caloric contents of the two beverages. The greater net gain in body protein following consumption of the HiEAA largely resulted from a greater suppression of protein breakdown, with a marginally greater stimulation of protein synthesis as compared to the LoEAA. Functional benefits of habitual consumption of the HiEAA would be expected to become evident over time, without adding significantly to daily caloric intake.

## Figures and Tables

**Table 1:** Baseline characteristics of all eight elderly women included in this cross-over study.

Age (year)	80 ± 7 ^a^
BMI (kg/m^2^)	32.7 ± 5.9
Body Weight (kg)	81.9 ± 16
Lean Body Mass (kg)	41.0 ± 7.0
Body Fat (%)	45.5 ± 3.2
Current Smoker (*n*, %)	0 (0%)
Systolic BP (mm Hg)	145 ± 17
Diastolic BP (mm Hg)	75 ± 5
Heart rate (beats/min)	73 ± 12
MMSE ^b^	28 ± 2
Medication (*n*, %) ^c^	
ACE inhibitor	4 (50%)
β-blocker	3 (37.5%)
Diuretics	6 (75%)
Angiotensin II receptor blockers	0 (0%)

^a^ mean ± SD, all such values. ^b^ MMSE, Mini-Mental State Examination. ^c^ Some subjects took a combination of medication subclasses. BMI, Body Mass Index; BP, Blood Pressure; ACE, Angiotensin Converting Enzyme.
